# The complete chloroplast genome sequence of *Spyridium parvifolium* var. *parvifolium* (family Rhamnaceae; tribe Pomaderreae)

**DOI:** 10.1080/23802359.2018.1483776

**Published:** 2018-08-13

**Authors:** Catherine Clowes, Rachael M. Fowler, Gillian K. Brown, Michael J. Bayly

**Affiliations:** a School of Biosciences, The University of Melbourne, Parkville, Australia;; b Department of Environment and Science, Queensland Herbarium, Toowong, Australia

**Keywords:** *Spyridium parvifolium*, Rosales, Rhamnaceae, *Ziziphus jujuba*, chloroplast genome

## Abstract

We assembled the complete chloroplast genome of the Australian shrub *Spyridium parvifolium* var. *parvifolium*. The genome was 161,012 bp in length, with a pair of inverted repeats (IRs) of 26,515 bp, separated by a large single copy (LSC) region of 88,814 bp and a small single copy region (SCC) of 19,168 bp. The GC content was 36.9%. In total, 130 genes were annotated, including 86 protein coding genes, 36 tRNA genes and 8 rRNA genes. Phylogenetic analysis of 56 chloroplast genes placed this genome of *S. parvifolium* var. *parvifolium* within the family Rhamnaceae.

*Spyridium* Fenzl is a member of the cosmopolitan Rhamnaceae family (VicFlora 2016), which includes the economically important Chinese Date, *Ziziphus jujuba* Mill. (Liu et al. 2014). *Spyridium parvifolium* (tribe Pomaderreae) is a shrubby, widespread, and morphologically variable species from south-eastern Australia (Jessop et al. 1986; Curtis and Morris 1993; VicFlora 2016; PlantNET n.d.). Several varieties of the species are sometimes recognized, and additional morphological variants have been identified (VicFlora 2016). Conflicting infraspecific taxonomies in different parts of Australia have implications for conservation management. In particular, two varieties (var. *parvifolium* and var. *mole*) are recognized in Tasmania, where both are listed as ‘Threatened’ under state legislation (Threatened Species Section 2016a, 2016b); in contrast, none of the varieties is currently recognized as distinct by the Australian Plant Census (CHAH 2016).

In this study, we report the complete chloroplast genome sequence of *S. parvifolium* var. *parvifolium* (GenBank accession MH234313). We generated this sequence to use as a reference in further chloroplast genome studies aimed at assessing phylogeography, genetic diversity, introgression, and infraspecific taxonomy of *S. parvifolium*.

Plant material was sampled from a population of var. *parvifolium* at Sisters Beach, Tasmania, Australia (40°54´15.0´´S 145°32´47.5´´E; Permit Number: TFL 15171; Voucher Specimen: MELUD155066a). Total DNA was extracted from leaves dried *in silica* gel using a modified CTAB protocol (Shepherd and McLay 2011), prepared for sequencing using the protocol of Schuster et al. (2018), and sequenced on an Illumina NextSeq 550 (mid-output, 2 × 150 Paired End kit) at The Walter and Eliza Hall Institute of Medical Research (WEHI). The genome was assembled by mapping paired reads to the reference genome of *Ziziphus jujuba* (accession number KU351660). Contigs built in Spades 3.10.0 (Bankevich et al. 2012), CLC Genomics Workbench 10.0.1 and Geneious 10.2 (Kearse et al. 2012) were mapped to the consensus sequence for quality control. Annotations were transferred from the reference sequence, with reading frames reviewed and manually adjusted.

The complete chloroplast of *S. parvifolium* var. *parvifolium* was 161,012 bp in length. A pair of inverted repeats (IRs) of 26,515 bp were separated by a large single copy (LSC) region of 88,814 bp and a small single copy region (SCC) of 19,168 bp. The GC content of the chloroplast genome was 36.9%. In total 130 genes were annotated, including 86 protein coding genes, 36 tRNA genes, and 8 rRNA genes. One pseudogene was predicted (*InfA*) and two truncated repeats were recorded at IR boundaries (*rps19* and *ycf1*). Annotations were identical between the reported genome (*S. parvifolium* var. *parvifolium*) and the reference (*Ziziphus jujuba*) except for one copy of the *ycf1* gene which was annotated on the reported genome as protein coding while neither copy of the gene was annotated as protein coding on the reference genome.

The phylogenetic tree presented in this study (Figure 1) builds from the results of Hauenschild et al. (2016) and Cheon et al. (2018). This tree shows *S. parvifolium* var. *parvifolium* within the Rhamnaceae clade and most closely related to *Ziziphus jujuba* (tribe Paliureae).

**Figure 1.**
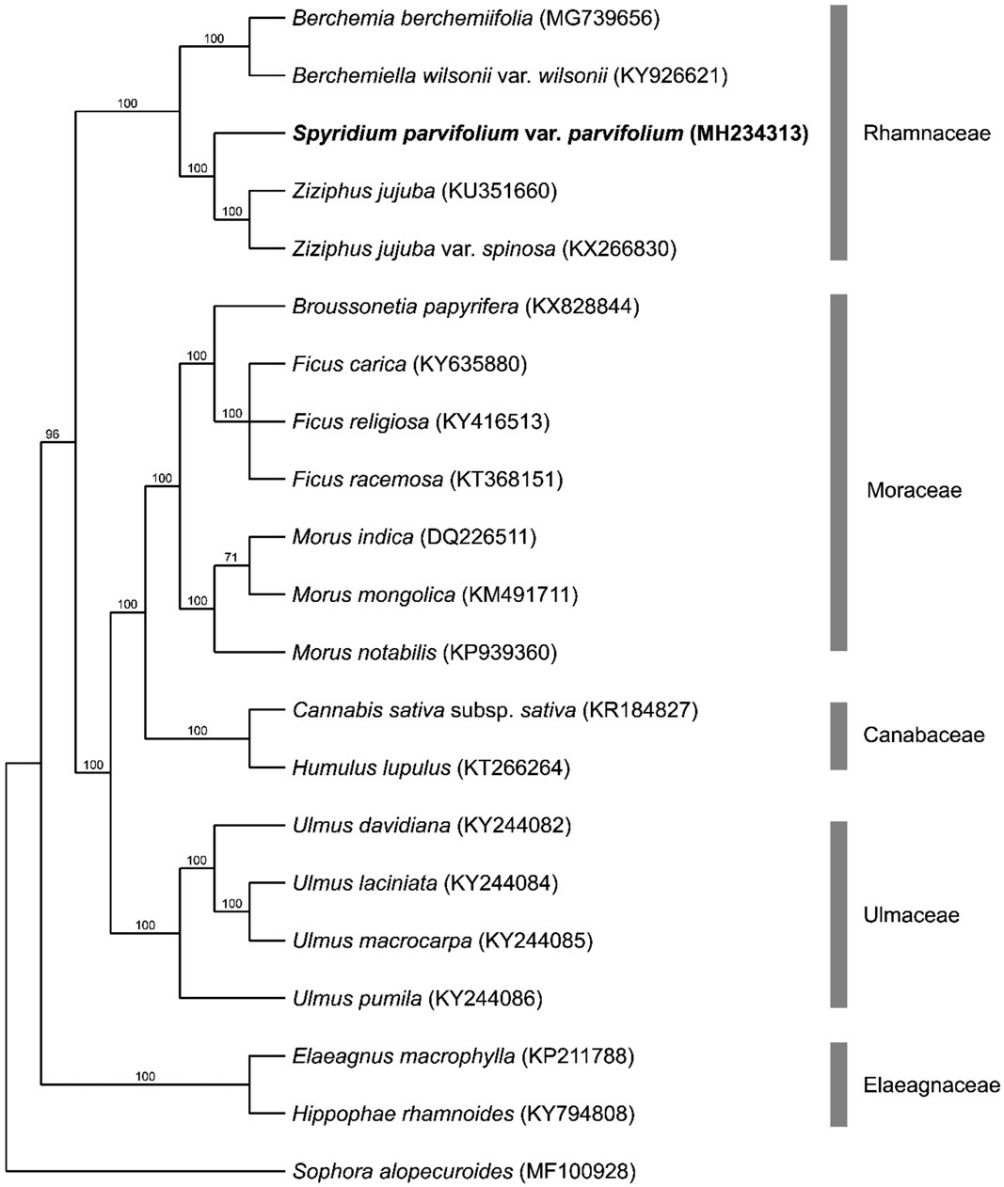
Bootstrap 50% majority rule consensus tree based on 56 protein coding chloroplast genes from 21 taxa including 20 species from the order Rosales and *Sophora alopecuroides* as the outgroup (CI = 0.8069 RI = 0.8744). Genes were aligned in MAFFT using default settings (Katoh et al. 2002). Sequences were analysed using maximum parsimony (MP) with PAUP 4.0a 161 using default settings (Swofford 2003). Bootstrap values are provided above branches. GenBank accessions are provided in brackets. *Spyridium parvifolium* var. *parvifolium* is highlighted in bold.
